# IFN-treated macrophage-derived exosomes prevents HBV-HCC migration and invasion via regulating miR-106b-3p/PCGF3/PI3K/AKT signaling axis

**DOI:** 10.3389/fcimb.2024.1421195

**Published:** 2024-10-28

**Authors:** Jing Chen, Qi Yin, Shiheng Xu, Xiaoqing Tan, Yu Liang, Chaohui Chen, Li Li, Tao Zhang, Tao Shen

**Affiliations:** 1 Department of Pulmonary and Critical Care Medicine, Yunnan Provincial Key Laboratory for Clinical Virology, Institute of Basic and Clinical Medicine, The First People’s Hospital of Yunnan Province, Kunming, China; 2 Medical School, Kunming University of Science and Technology, Kunming, China; 3 Faculty of Life Science and Technology, Kunming University of Science and Technology, Kunming, China; 4 Department of Infectious Diseases and Hepatic Disease, Yunnan Province Innovation Team of Intestinal Microecology Related Disease Research and Technological Transformation, The First People’s Hospital of Yunnan Province, Kunming, China

**Keywords:** IFN-α-induced macrophage-derived exosome, miR-106b-3p, PCGF3, HBV-related hepatocellular carcinoma, PI3K/AKT pathway

## Abstract

**Background:**

Studies revealed that exosomes from IFN-α-treated liver non-parenchymal cells (IFN-exo) mediate antiviral activity. MiR-106b-3p has been shown to play a paradoxical role in disease progressing from different studies. However, its specific role in HBV-related hepatocellular carcinoma (HBV-HCC) and the underlying mechanism remains unclear.

**Method:**

Huh7 cells transient transfected with plasmids of HBV-C2 and B3 were co-cultured with IFN-exo. Cell supernatants were collected to detect miR-106b-3p, HBsAg, HBeAg and HBV DNA levels. Cell proliferation, apoptosis, migration and invasion were analyzed. The putative targets of miR-106b-3p were identified by a dual-luciferase reporter system. The expression of PCGF3, migratory proteins(MMP2/9), and the PI3K/AKT signaling pathway-related proteins were assessed by western blot. The expression of PCGF3 mRNA was quantitative analyzed by using 52 pairs of paraffin-embedded tissues from HCC patients. siRNAs-PCGF3 were used to knocked-down PCGF3 expression.

**Results:**

The expression of miR-106b-3p was significantly higher in THP-1 cells and supernatants treated with IFN-exo than those untreated. Significantly increased expression of miR-106b-3p and decreased expression of HBsAg and HBV DNA were observed in Huh7-C2/B3 cells treated with IFN-exo. In addition, miR-106b-3p was directly target to PCGF3. Scratch healing assay and transwell assay showed that either IFN-exo or miRNA-106-3p over-expression, or siRNAs-PCGF3 inhibited migration and invasion of Huh7-C2/B3 cells, and subsequently resulted in suppression of p-AKT/AKT and p-PI3K/PI3K. Notably, the expression level of PCGF3 was significantly lower in HBeAg (+)-HCC tumor tissues than HBeAg (-)-HCC tumor.

**Conclusion:**

IFN-α-induced macrophage-derived miR-106b-3p inhibits HBV replication, HBV- Huh7 cells migration and invasion via regulating PCGF3/PI3K/AKT signaling axis. miR-106b-3p and PCGF3 were potential biomarkers in the prevention and treatment of HBV-HCC.

## Introduction

Hepatitis B virus (HBV) infections can result in asymptomatic hepatitis, acute viral hepatitis B, chronic viral hepatitis B, cirrhosis, decompensated cirrhosis, severe hepatic failure, and hepatocellular carcinoma (HCC). HCC is one of the most common cancers, and its incidence worldwide has steadily increased. HBV is an important pathogen causing HCC. Due to HBV’s aberrant replication mechanism, which involves the reverse transcription via RNA intermediate and, as well as the lack of proofreading capability of its viral polymerase, HBV exhibits a high degree of sequence heterogeneity, thus, based on the differences in its whole gene sequences, it is classified into different genotypes and subgenotypes (Kao, 2011; Liu et al., 2021; Chen et al., 2023; Sant’Anna and Araujo, 2023). Depending on the specific HBV genotype, the prognosis, immune response, clinical symptoms, and response to antiviral drugs vary (Hadziyannis, 2011; Chen et al., 2023). Currently, the main anti-HBV therapeutic drugs used in clinical practice are interferon (IFN)-α and nucleoside (nucleotide) analogs (NA). The antiviral effect of IFN-α can show viral (sub)genotype-dependent differences (Zhang et al., 2021). Moreover, IFNs inhibit HBV-HCC progression by decreasing the stability of HBV pgRNA. As an adjutant to the curative treatment of HBV-related HCC (HBV- HCC), IFNs reduces the mortality rate of patients (Zhang et al., 2014). Research indicates that IFNs exert antiviral effects upon liver non-parenchymal cells (LNPC) secreted exosomes to virus-infected liver cells (Li et al., 2013). However, it is unclear whether the antiviral effects, delivered via exosomes, are also subject to HBV genotype-dependent differences.

Covered in a phospholipid bilayer membrane, exosomes contain various cellular components, such as proteins, microRNAs (miRNAs), long-chain non-coding RNAs, circular RNAs, messenger RNAs, and DNA. Existing studies have shown the involvement of miRNAs in the development and progression of HCC. MiRNAs are endogenous small non-coding single-stranded RNAs, which regulate the expression of downstream genes by complementary binding with the 3’-untranslated regions of target mRNAs. Increasing evidence suggested that miRNAs were involved in regulating different biological processes, such as cancer cell proliferation, apoptosis, migration, and invasion. Furthermore, they were associated with disease progression such as gastric cancer, non-small cell lung cancer (NSCLC), cardiovascular disease, breast cancer, HCC, and HBV-HCC. For example, miR-342-3p, a tumor suppressor with low expression among HCC patients, delayed tumor progression and improved survival in HCC mouse models (Komoll et al., 2021). Exosome-derived miR-5a-3p inhibited the proliferation, migration, and invasion of HCC cells (Lin et al., 2020). In addition, using microRNA microarray technology, 94 plasma samples and 13 liver biopsies from IFN-treated chronic hepatitis B patients revealed that miR-106b may be one of the early IFN-responsive miRNAs (Zhang et al., 2012). The aberrant expression of miR-106b-3p was reported in several tumors. It may be involved in cell migration, invasion, and epithelial-mesenchymal transition, and it was correlated with prognosis (Qiao et al., 2019; Liu et al., 2020). A multicenter study on HCC showed that circulating miR-106b-3p were viable diagnostic biomarkers for HCC, and its expression in the plasma of cirrhotic patients was significantly lower than that of HCC patients (Moshiri et al., 2018). However, it is rarely reported in HBV-related HCC.

As a homolog of the polycomb group ring finger proteins 1/2/4/5/6, the polycomb group ring finger protein PCGF3 mediates epigenetic histone modifications and participates in the regulation of chromosome structure, X chromosome silencing, and gene transcription. Reportedly, the aberrant expression and mutations of PCGF3 were related to drug efficacy, diagnosis, and progression of various diseases (Almeida et al., 2017; Chen et al., 2021). Specifically, PCGF3 promotes the proliferation and migration of NSCLC cells by regulating the PI3K/AKT signaling pathway (Hu et al., 2021). However, its role in HBV-infected diseases and liver diseases remains unclear. Existing literature reports that DLC-1, NFRSF21, ACPL2, EPHA5, RAB22A, SLITRK3 and other genes were miR-106b-3p-regulated target genes (Lu et al., 2014; Chen et al., 2023). In this study, PCGF3 was identified as a possible target gene of miR-106b-3p using the TargetScan software. However, there have been no reports on the regulation of PCGF3 by miR-106b-3p.

This study was carried out using HBV-HCC cells and tissues from HCC patients. The results showed that IFN-treated macrophage-derived exosomes (IFN-exo) highly expressed miR-106b-3p. Moreover, it inhibited the replication of the Huh7-C2 and B3 subgenotype virus and the expression of hepatitis B surface antigen (HBsAg). Differences in the response of Huh7-C2/B3 were noted. In addition, miR-106b-3p was an IFN-responsive miRNA that directly targeted PCGF3, and it inhibited the migration and invasion of HBV-positive Huh7 cells by modulating the PI3K/AKT signaling pathway. PCGF3 was also associated with a poorer prognosis in HBV-positive patients. Therefore, they are potentially important biomarkers for the treatment and prevention of HBV.

## Materials and methods

### Cell culture and transfection

Human hepatoma cell line Huh7, HepG2, HepG2.2.15 were purchased from the BeNa Culture Collection (Henan, China) and authenticated by short tandem repeat (STR) markers. Huh7 was maintained in DMEM medium (GIBCO, China, USA), supplemented with 10% fetal bovine serum (vol/vol) (FBS; Biological Industries, CT, USA), 1% penicillin, 1% streptomycin and 1% gentamicin. 4× 10^5^ Huh7 cells were plated into each 6 -well plates and incubated overnight till 80-90% confluent. 2 μg of chemical synthetized 1.3 mer replication-competent recombinant plasmids pcDNA3.1 (+)/HBV (nt1039-3215-1986) for HBV-B3 (Accession No.KP148458) and HBV-C2 (Accession No. KP148352) subgenotypes were transfected separately into Huh7 cells using the TurboFect transfection reagent (Thermo, United States), in triplicate and incubated at 37°C with 5% CO_2_. The supernatant was collected on 24h, 48h and 72h post-transfection. HBsAg and HBeAg were tested by Elisa (Shanghai Kehua Bio-Engineering Co., Ltd, China) and HBV DNA was quantified by qRT-PCR (Zhuhai Livzon Pharmaceutical Group Inc., China) according to the manufacture’s instruction.

THP-1, LO2 and HEK293T cells were purchased from Shanghai Cell Bank (China) and authenticated by STR markers. Cells were maintained in RPMI-1640 medium (GIBCO, China, USA) with 10% (vol/vol) FBS, 1% penicillin, 1% streptomycin and 1% gentamicin. THP-1 monocytes were treated with phorbol 12-myristate 13-acetate (PMA) at concentration of 50 ng/ml for 24h to obtain a macrophage-like phenotype. Then, removed the medium and added fresh medium with 10% Exosome Depleted Fetal Bovine Serum (ViVaCell, Shanghai XP Biomed Ltd., China), with or without 3000 U/ml IFN-α (Beijing Tri.prime.gene) for 48 h, exosomes were purified from the culture supernatant by differential centrifugation as previously described (Yao et al., 2019). Purified exosomes were then characterized by western blot and electron microscopy as previously described (Tang et al., 2017).Tumor susceptibility gene 101 (TSG101) and CD9 antigen were used as markers to identify the purity exosomes (Raab-Traub and Dittmer, 2017; Yao et al., 2018; Yang et al., 2023).

### Cell-co-culture

Huh-7 cells 72h post-transfected with pcDNA3.1(+)-HBV-C2/B3 were treated with indicated reagents using lipofectamine 3000 (Thermo,USA). In brief, Huh-7 cells with or without HBV were transfected with either IFN-exo (10ug/ml final concentration), or miR-106b-3p mimic, or control mimics (75nM final concentration)(HanBio Co., Ltd., Shanghai, China), or siRNAs-PCGF3, or si-PCGF3-NC (75nM final concentration) (GenePharma Co., Ltd., Suzhou, China), or LY294002(15μM final concentration)(MCE, HY-10108)and co-cultured for 48 h, then, cells and supernatant were collected to the further analysis. Sequences of above mentioned siRNAs were listed in the **Supplementary Table 1**. The siRNA(s) with strongest PCGF3 silencing effect was (were) used to the rescue experiments.

### Luciferase reporter assay

The 3’-UTR sequence of PCGF3 predicted to interact with miR-106b-3p, together with a corresponding mutated sequence within the predicted target sites, were synthesized and inserted into the pSI-Check2 dual-luciferase miRNA target expression vector called PCGF3-WT and PCGF3 -MUT. Subsequently, HEK293T cells were seeded into a 96-well plate and co-transfected with wild-type or mutant 3’-UTR of PCGF3 vector and miR-106b-3p mimic or mimic NC using the Lipofectamine 3000 reagent. After 48 h, cells were harvested and luciferase activity was measured according to the manufacturer’s instructions (Dual-Luciferase Assay System; Vigorous Biol. Beijing). Renilla luciferase activity was normalized to Firefly luciferase.

### Cell proliferation analysis

Cell viability and proliferation was assayed by CCK8-kit (MCE, HY-K0301-3000T, USA) according to the manufacturer’s instructions. In brief, Huh7 cells were seeded into 96-well plates at a density of 2×10^4^ cells/well till the concentration reached about 70% and transfected with the recombinant plasmids of HBV-C2/B3 for 24h, followed by incubation with miR-106b-3p mimic/NC or IFN-exo. 10 μL CCK-8 reagents was added into each well in gradient times (0,24,48,72 and 96h) and cultivated for 2h at 37°C in the dark before measuring the absorbance of mixtures at 450 nm wavelength using a microplate reader (BioTek SYNERGY H1, USA). Each group was performed with three repetitions.

### Cell apoptosis analysis

The Huh7 cells treated with indicated reagents were harvested and washed twice with cold phosphate buffer saline (PBS). The apoptosis rate was evaluated using the Annexin V-fluorescein isothiscyannate (FITC)/propidium iodide (PI) kit (Solarbio, CA1020, China) according to the manufacturer’s instructions. In brief, the 100µl cell suspension were stained with 5µl Annexin V/FITC for 5 minutes at room temperature in dark, then added 5µl of PI and 400µl of PBS and evaluated using a BECKMAN Flow Cytometer (DxFLEX, Beckman Coulter, China). The rate of apoptosis was analyzed using FlowJo software (v10.0). This experiment was repeated at least three times.

### Cell migration analysis

The scratch wound healing assay was used to evaluate cell migration. The Huh7 cells 72h post-transfected with or without HBV-C2/-B3 plasmids were incubated in a 6-well culture plate with treated reagents of replicate (3 wells), and placed at 37 °C, 5% CO_2_ for 24 h. Remove the medium and scratch the surface of the inoculated cells with a pipette tip and mark it. Wash gently once with PBS. Photographed the scratches at 0 h, 24 h, 48 and 72h under a microscope (OLYMPUS CKX53, U-CTR30-2), and the distance that the cells migrated to the wounded area during this time was measured with ImageJ Software (V1.53, Media Cybernetics, Silver Springs, MD, USA). All experiments were performed in triplicate.

### Cell invasion analysis

The transwell assays was used to evaluate cell invasion. 100 µl matrigel mixture (mixed with Matrigel and DMEM 1:7) was pre-coated in the upper layer of the chamber (12 μm pore size, Labselct). Then, the Huh7 cells 48h post-transfected with or without HBV-C2/-B3 plasmids were collected and 1.75×10^6^ cells/ml cells were resuspended in 200 µl serum-free medium and added on the top of the transwell membrane in the upper chamber of replicate (3 wells). 600 µl DMEM medium containing 10% FBS was added to the lower chamber. After 24h incubation with treated reagents, cells were fixed in 4% paraformaldehyde for 30 min, and stained with 1% crystal violet for 30 min. After removing the cells on the inner layer, 10 randomly views were photographed under a microscope (OLYMPUS CKX53, U-CTR30-2), and average cell number per view was counted with ImageJ software. All experiments were performed in triplicate.

### Quantitative reverse transcriptase polymerase chain reaction

Total RNA from the cultured cells or paraffin-embedded tissue of HCC patients’ was extracted using RNAsimple Total RNA Kit (TIANGEN, DP419) or RNeasy FFPE Kit (Qiagen, 73504) according to the manual instructions, respectively. For mRNA, cDNA was reverse transcribed using Fasting RT kit (with gDNase) (TIANGEN,KR116), and relative mRNA levels were quantitated using SuperRealPreMix Plus (SYBR Green) (TIANGEN,FP205) and normalized to the house keeping gene GAPDH. For miRNA, cDNA was reverse transcribed using miRcute Plus miRNA First-Strand cDNA kit (TIANGEN, KR211), and relative miRNA levels were quantitated using miRcute Plus miRNA qRT-PCR kit (SYBR Green) (TIANGEN, FP411) and normalized to the uniformly expressed U6 snRNA. qRT-PCR was performed on a gene amplication System (Roche LightCycler480, Switzerland)by the below procedure (95°C, 3 min; 94°C 10s, 64°C 20s, 45 cycles). All qRT-PCR amplification was performed in triplicate and repeated in three independent experiments. The quantification results were analyzed by the relative quantitation 2-ΔΔCT method.

### Immunoblot analysis

The total proteins were lysed using RIPA Lysis Buffer(Biosharp, BL504A)supplemented with protease and phosphatase inhibitors (Thermo) and quantified with BCA Protein Assay Kit (Tiangen, Beijing, China). Proteins were loaded on SDS-PAGE gel and separated, and then transferred onto PVDF membranes (Sigma). The membranes were blocked with 5% nonfat milk in TBST for 2 h at room temperature or overnight at 4°C and incubated with specific primary antibodies overnight at 4°C (Information of the antibodies were listed in **Supplementary Table 1**). Then the membranes were washed three times using TBST and incubated with HRP conjugated secondary antibody for 2 h at room temperature (SolarBio, China). Detection was performed by enhanced chemiluminescence kit (MCE, USA) and proteins were visualized on the Bio-Rad ChemiDoc XRS Imager system (Bio-Rad Laboratories, Berkeley, CA, USA).

### Bioinformatics analysis

To elucidate the molecular mechanisms by which miR-106b-3p exerts its functional effects on HCC cells, target genes that potentially bind to miR-106b-3p were predicted using TargetScan software (https://www.targetscan.org/vert_80/).To predicted the clinical value in silico, expression profile and Kaplan-Meier survival analysis of HCC patients for potential target genes of miR-106b-3p were determined by using UALCAN online database (https://ualcan.path.uab.edu/).

### Clinical tissues

Paraffin-embedded tissues and matched adjacent non-tumor tissues were obtained from 190 patients who received surgery from January 2019 to July 2023 at the First People’s Hospital of Yunnan Province (Kunming, China).Patients who received chemotherapy or radiotherapy before surgery were excluded. 52 patients were finally selected to detect the expression of PCGF3 mRNA. Among these patients, 9 pairs with HBV negative and 43 pairs with HBV positive. Written informed consents were obtained from all patients. This study was approved by the Ethics Committee of the First People’s Hospital of Yunnan Province (KHLL2023-KY162).

### Statistical analysis

Statistical analyses were executed using GraphPad Prism 9 (V9.0,GraphPad Software Inc., La Jolla, CA, USA) and IBM SPSS Statistics software (SPSS, Inc., Chicago, IL, USA) (Ver.26), with *P <* 0.05 indicating statistical significance. Statistical significance depending on structure of the data, one-way or two-way ANOVA (Multiple comparisons), a two-tailed Student t-test, Pearson’s correlation analysis, Kaplan-Meier method and the log-rank test were used to evaluate the statistical significance. The measurement data are deviation from at least three independent experiments.

## Result

### IFN-induced macrophage-derived exosomes can up-regulate the expression level of miR-106b-3p in Huh7-HBV (+) cells

**Figure 1:**
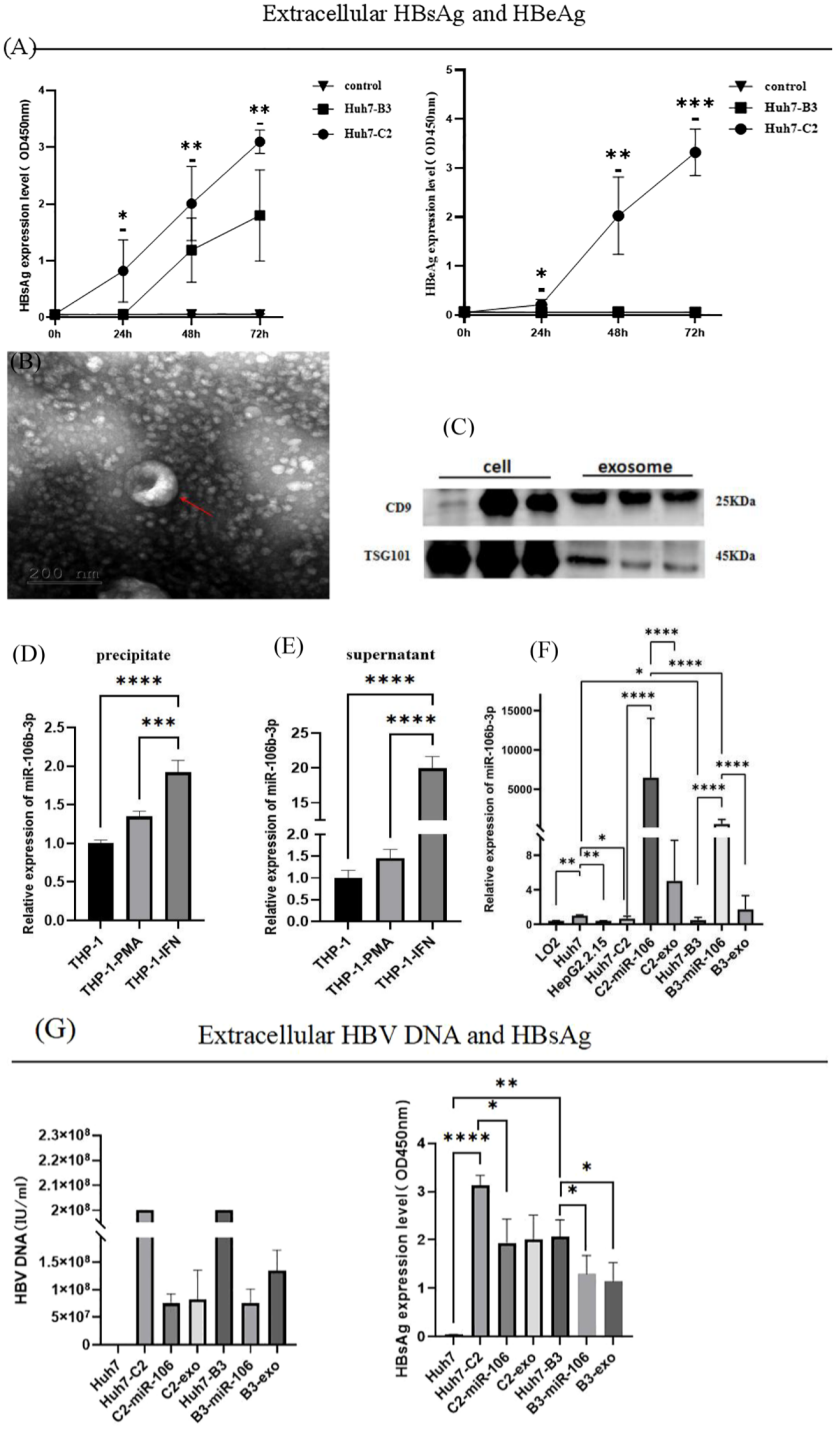
Expression of miRNA-106b-3p, HBsAg, HBeAg and HBV DNA in Huh7-HBV- C2 and B3 cells after co-cultured with interferon-induced macrophage-derived exosomes. **(A)** The extracellular expression of HBsAg and HBeAg were determined from Huh7 cells that were transfected with 1.3 mer HBV DNA containing HBV-C2 or HBV-B3 subgenotype, with supernatant been collected on 24h, 48h and 72h post-transfection (* represents a statistical difference of Huh7-C2 in comparison with Huh7-B3). **(B)** Electron microscopy of purified exosomes from IFN-α-treated macrophages. Scale bar, 200 nm. **(C)** Immunoblot analysis of exosomal markers in exosomes prepared from IFN-α-treated macrophages and in the corresponding whole-cell lysates. **(D, E)** The expression of miR-106b-3p in exosomes prepared from IFN-α-treated macrophages and in the corresponding whole-cell lysates. **(F)** The expression of miR-106b-3p in liver cell lines untreated or treated with IFN-treated macrophages-derived exosomes or miR-106b-3p mimic. **(G)** The extracellular expression of HBV DNA and HBsAg were determined from HCC cells untreated or treated with IFN-α-treated macrophages-derived exosomes miR-106b-3p mimic (The DNA levels of Huh7-C2 and B3 were over the highest detection limit). (*P < 0.05, **P < 0.01, ***P < 0.001, ****P < 0.0001).

After transfection of recombinant HBV-C2 and HBV-B3 plasmids in the Huh7 cell line respectively, the expression of HBsAg and hepatitis B e-antigen (HBeAg) was detected over time. The Huh7-C2 subgenotype had a significantly higher HBsAg expression than the Huh7-B3 subgenotype. The expression of HBeAg was only detected in the HBV-C2 genotype within 72 h (**Figure 1A**). After IFN-α stimulation of THP-1, exosomes were obtained from the supernatant via ultra-high-speed centrifugation, and electron microscopy revealed cup-shaped microvesicles with a diameter of approximately 30-150 nm (**Figure 1B**). On Western blot, the specific surface and membrane marker molecule of exosome, CD9 and TSG101 were detected (**Figure 1C**). The qRT-PCR results showed that the THP-1 cell precipitates and supernatant in the IFN-α treatment group exhibited a significantly higher miR-106b-3p expression than the THP-1 cells untreated with IFN-α (**Figures 1D, E**). The expression of miR-106b-3p was significantly lower in LO2 cells, HepG2.2.15 cells, and Huh7-C2 and B3 cells, compared to Huh7 HCC cells. After co-culturing IFN-exo or miR-106b-3p-mimic with HBV-positive Huh7 cells for 48 h (**Figure 1F**), the expression of miR-106b-3p in Huh7-C2 and B3 cells was significantly higher, while the expression of HBsAg and the HBV DNA level in the culture supernatant were significantly decreased (**Figure 1G**).

### PCGF3 is a direct target of miR-106b-3p

**Figure 2:**
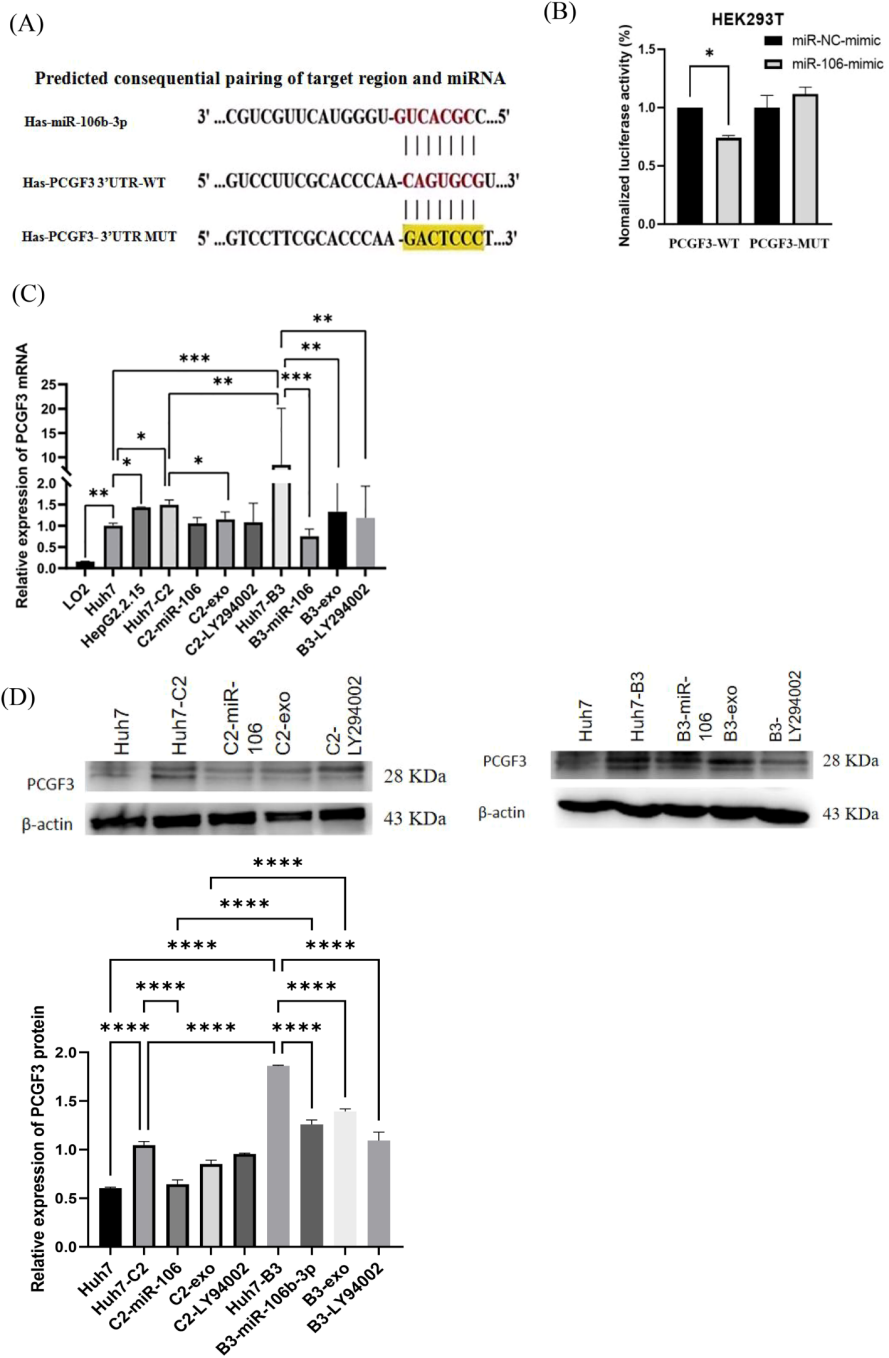
PCGF3 is a direct target of miR-106b-3p. **(A)** The wild-type (WT) and mutant (MUT) binding sites of the PCGF3 3′-UTR to miR-106b-3p were cloned into the pSI-Check2 vector. **(B)** miR-106b-3p overexpression significantly suppressed the luciferase activity that carried wild-type (wt) but not mutant (mut) 3′-UTR of PCGF3. **(C, D)** miR-106b-3p mimic and IFN-exo can significantly down-regulate the expression of PCGF3 in Huh7 cells with HBV C2 or B3, as determined by qRT-PCR and western blotting All data were expressed as mean ± SD. (*P < 0.05, ** *P <* 0.01, ***P < 0.001, ****P < 0.0001).

As one of the predicted target genes of miR-106b-3p by using TargetScan analysis, PCGF3 was selected to be further studied. The predicted WT- and MUT2- of PCGF3 3′-UTR binding sites to miR-106b-3p were cloned into the pSI-Check2 vector (**Figure 2A**). Then, luciferase reporter assay confirmed that miR-106b-3p over-expression significantly decreased the luciferase activity of WT-PCGF3 3′-UTR, but no significant effect on the luciferase activity of MUT2-PCGF3 3′-UTR (**Figure 2B**). To confirm this hypothesis, we performed qRT-PCR analysis and found that PCGF3 were highly expressed in HBV C2 and B3 compared to Huh7 cells (P<0.01), whereas both IFN-α treated macrophage-derived exosome and miR-106b-3p mimic can dramatically decrease the expressions of PCGF3 mRNA (**Figure 2C**) and protein (**Figure 2D**) in Huh7-C2 and B3 cells.

### Clinical significance of PCGF3 expression for HCC patients

**Figure 3:**
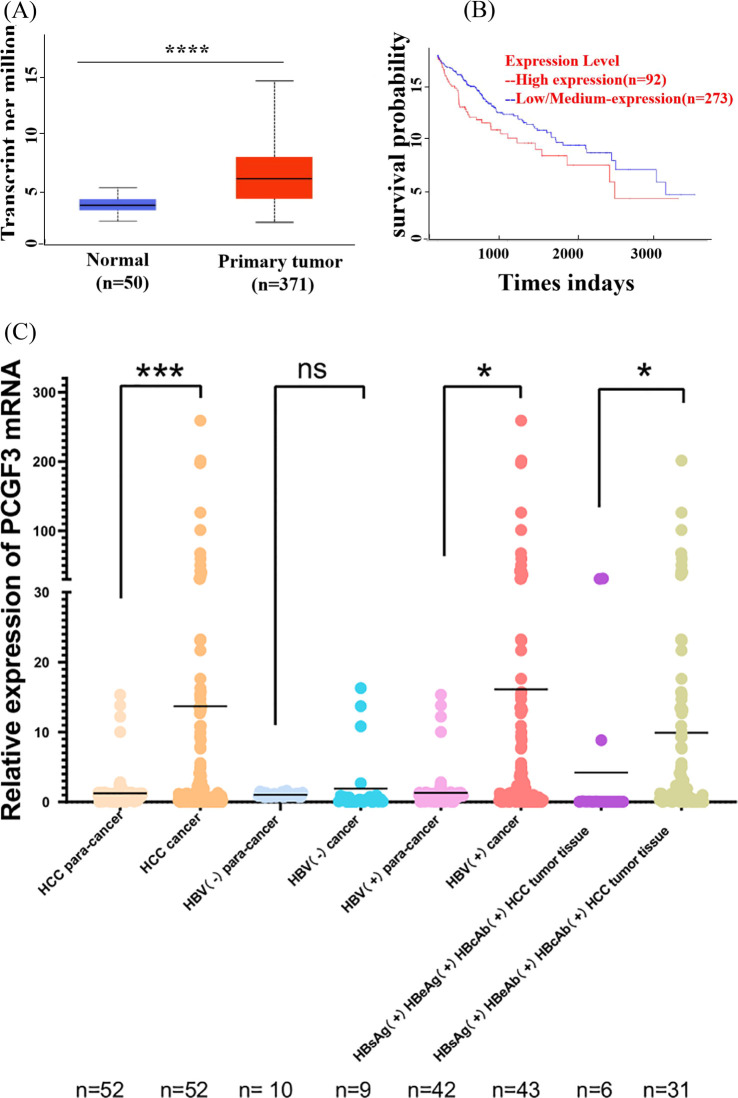
The prognostic value of PCGF3 for HCC patients. **(A)** Expression level of PCGF3 in normal and tumor tissues from HCC patients on TCGA database. **(B)** Overall survival (OS) was compared between PCGF3 high expressing HCC patients and low expressing patients. **(C)** Expression level of PCGF3 in tumor tissues and adjacent tumor tissues from non-HBV-HCC and HBV-HCC patients.(All data were expressed as mean ± SD. (*P < 0.05, ***P < 0.001, ****P < 0.0001, ns. not significant).

Data from UALCAN online database showed that the expression level of PCGF3 was significantly higher in HCC tumor tissues than in normal tissues (**Figure 3A**). In addition, Kaplan-Meier survival curves suggested that the overall survival of patients with high PCGF3 expressing was significantly worse (*P <* 0.05) (**Figure 3B**), suggesting that PCGF3 is associated with a dismal prognosis for HCC patients. Moreover, HCC or HBV-HCC tumor tissues were with significantly higher expression level of PCGF3 than that in matched adjacent non-tumor tissues, HBsAg(+)/HBeAb(+)/HBcAb(+) HCC tumor tissues were with significantly higher expression level of PCGF3 than that in HBsAg(+)/HBeAg(+)/HBcAb(+) HCC tumor tissues, and males were more susceptible progression to HBV-related HCC compared to females (**Figure 3C** and **Table 1**).

**Table 1:** Correlation of PCGF3 expression with clinical characteristics.

Characteristic	HBV positive(n=43)	HBV negative(n=9)	p-value
Gender	43	9	0.757
Male	38	7	<0.001^a^
female)	5	2	0.180^b^
Age, median years (range)			0.295
<60 (n=38)	50 (25-59)	55 (23-59)	0.688
≥60 (n=14)	65 (60-79)	72 (66-79)	0.099
HBsAg (+) HBeAg (+) HBcAb (+)	6		<0.001^a^
HBsAg (+) HBeAb (+) HBcAb (+)	31		

a *P* value of Clinical characteristics comparison among HBV positive patients.

b *P* value of Clinical characteristics comparison among HBV negative patients.

### IFN-induced macrophage-derived exosomes or miR-106b-3p inhibits HCC cell growth, migration and invasion via down-regulate PCGF3 expression *in vitro*

**Figure 4:**
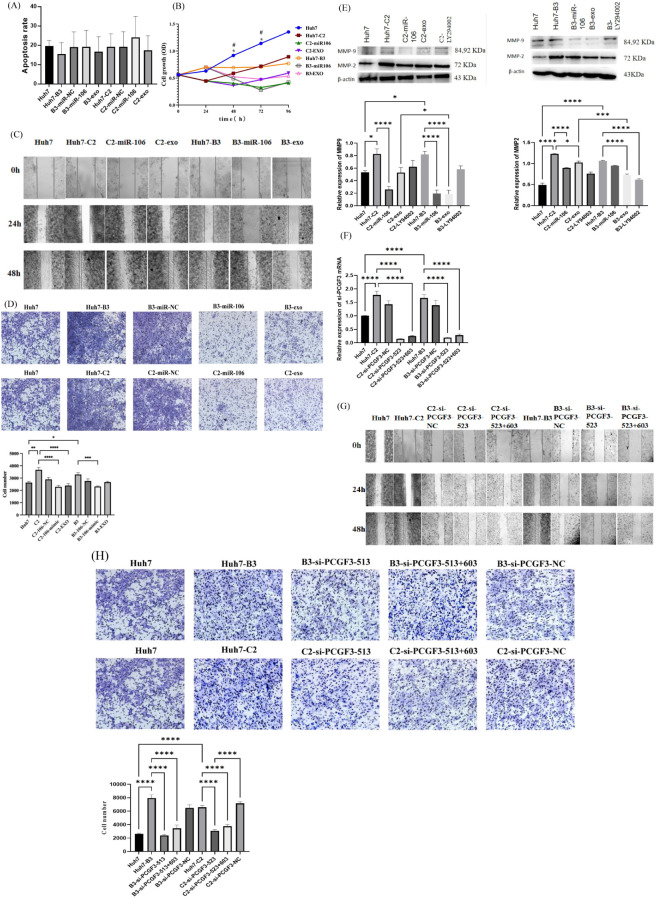
The effect of IFN-α-treated macrophages-derived exosomes (IFN-exo) and miR-106b-3p on PCGF3 expression and Huh7-HBV(+)cells function. **(A)** IFN-exo and miR-106b-3p Annexin V-FITC staining showed no obvious effect on apoptosis of Huh7-C2 and B3 cells untreated or treated with IFN-exo or miR-106b-3p. **(B)** IFN-exo and miR-106b-3p inhibited the proliferation of Huh7-C2 and B3 cells (*represents a statistical difference between Huh7-C2 untreated and treated with miR-106b-3p; # represents a statistical difference between Huh7-B3 untreated and treated with miR-106b-3p). **(C, D)** Scratch wound healing assay and transwell assays revealed that HBV-C2 and B3 positive promoted migration and invasion of Huh7 cells, while IFN-exo and miR-106b-3p abolished the effects of HBV positive on Huh7 cells. **(E)** Western blot analysis indicated that MMP-9 and MMP-2 were higher expression in HBV-C2 and B3 positive Huh7 cells than Huh7 cells, while IFN-exo, miR-106b-3p and LY294002 reduced the expression of MMP-9 and MMP-2 in HBV-C2 and B3 positive Huh7 cells. **(F–H)** siRNAs-PCGF3 knocked-down PCGF3 mRNA expression and inhibited migration and invasion of HBV-C2 and B3 positive Huh7 cells. All data were expressed as mean ± SD. (*, #*P <* 0.05, ** *P <* 0.01, *** *P <* 0.001, ****P < 0.0001).

To investigate the biological function of miR-106b-3p and PCGF3 in HCC, gain-and loss-of-function experiments were performed in Huh7 cells with or without HBV-C2 and HBV-B3, respectively. As measured by qRT-PCR, PCGF3 was effectively down-regulated in Huh7 cells treated with miR-106b-3p mimic or IFN-exo (**Figures 2C, D**). Cell apoptosis analysis showed no significant effect on Huh7 HBV (-)/HBV (+) cells after modulating miR-106b-3p expression (**Figure 4A**). However, cell proliferation, scratch test and transwell assays revealed that miR-106b-3p and IFN-exo significantly inhibiting the proliferation (**Figure 4B**), migration (**Figure 4C**) and invasion (**Figure 4D**) of Huh7-HBV- C2/B3 cells, and reducing the expression of MMP-2 and MMP-9 (**Figure 4E**); Whereas PCGF3 knockdown by transfection with si-PCGF3 (**Figure 4F**) also significantly inhibited the migration and invasion of Huh7- C2/B3 cells (**Figures 4G, H**).

### IFN-induced macrophage-derived exosomes or miR-106b-3p down-regulates PCGF3 expression and inhibits PI3K/AKT pathway in HCC cell

**Figure 5:**
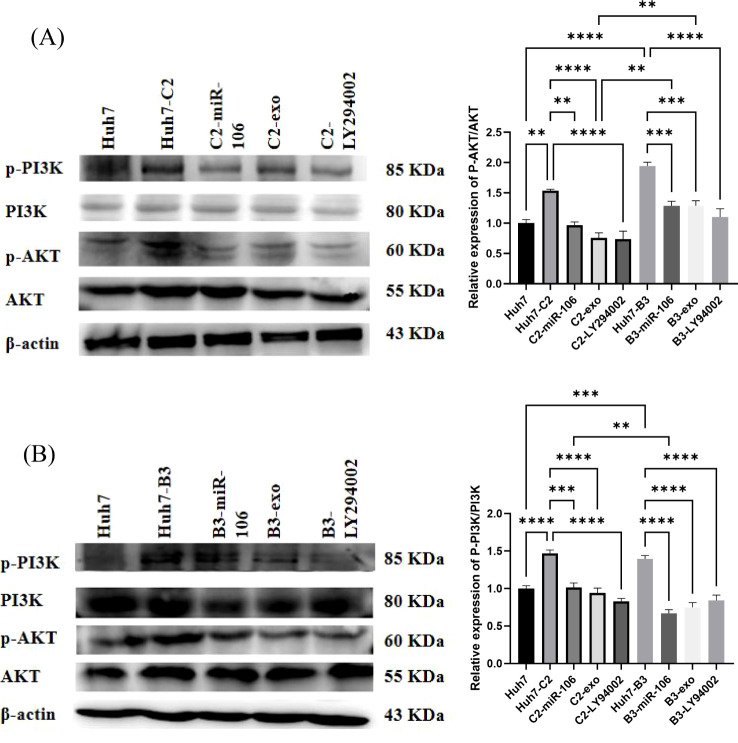
The effect of IFN-α-treated macrophages-derived exosomes (IFN-exo), miR-106b-3p or LY294002 on PI3K-AKT signaling pathway. **(A)** Western blot analysis indicated that IFN-exo, miR-106b-3p and LY294002 inhibited the activity of p-AKT. **(B)** Western blot analysis indicated that IFN-exo, miR-106b-3p and LY294002 inhibited the activity of p-PI3K. (** *P <* 0.01, ***P < 0.001, ****P < 0.0001).

The aforementioned results showed that IFN-exo and miR-106b-3p-mimic can significantly reduce the expression of PCGF3. Studies have shown that PCGF3 promoted the proliferation and migration of NSCLC cells by regulating the PI3K/AKT signaling pathway (Hu et al., 2021). To unveil underlying mechanisms, the activity of the PI3K/AKT signaling pathway was also examined in this study. The qRT-PCR results showed that after treatment with the pathway inhibitor LY294002, the expression of PCGF3 was significantly reduced in Huh7-B3 cells and 27.4% reduced in Huh7-C2 cells (**Figures 2C, D**). The western blot results showed that the PCGF3 protein levels, p-AKT/AKT, and p-PI3K were significantly higher in Huh7-C2 and B3 cells than in Huh7 cells (**Figures 5A, B**). Additional treatment with IFN-exo, miR-106b-3p-mimic, and LY294002 significantly reduced the PCGF3 protein levels as well as those of p-AKT/AKT and p-PI3K/PI3K in Huh7-C2 and B3 positive cells (**Figures 5A, B**).

## Discussion

HBV infection is one of the most important risk factors for the development and progression of HCC. As genetically heterogeneous viruses, different HBV (sub) genotypes present with differences in the onset and progression of HBV-associated diseases, drug response and viral load persistence, and viral antigenic shift. The predominant genotype in Asia is the HBV-C genotype, followed by the B genotype (Bello et al., 2023; Chen et al., 2023). The intergenotypic recombination between HBV genotype B and C are predominantly recombinant event worldwide (Araujo, 2015), HBV B3 is one of the recombinant isoforms between B and C (Nurainy et al., 2008; Qian et al., 2015). Patients with the HBV-C genotype experience a more severe type of liver injury (Shi, 2012). Moreover, the prevalence of HCC is higher among patients with the HBV-C genotype than those with the HBV-B genotype (Wang et al., 2019). Primary human liver cells infected with HBV genotype C1 exhibited the poorest drug response when treated with IFN-alpha2a or the entecavir, while those infected with genotype B2 had the greatest reduction in HBV DNA. Compared to the genotypes C2, B1, and A2, the subgenotype F1b had the highest expression of both HBsAg and HBeAg levels (Zhang et al., 2021; Fernandes da Silva et al., 2023), genotype D1 had the lowest level of HBsAg, and genotype C1 had the highest level of HBeAg (Elizalde et al., 2021). However, serum HBV DNA levels of HBV patients with B/C intergenotypic recombinants were conflicted, which may lower (Liao et al., 2017)or higher than those without intergenotypic recombinant virus (Liu et al., 2018) based on different reports. The results of the present study also showed that there were differences in antigenic expression among different subgenotypes. After the transfection of Huh7 cells with a recombinant HBV-C2 and B3 plasmids, the HBV-C2 subgenotype expressed HBsAg earlier than the HBV-B3 subgenotype, and HBeAg was only detected in the C2 genotype within 72 h. In fact, the B3 subgenotype used in this study was a wild-type strain without the stop codon mutation in the pre-core region. Theoretically, it can express HBeAg, but HBeAg was not detected via ELISA assay at 72 h, which suggested that the HBV-B3 strain used in this study was a viral strain with a low expression for HBeAg. In addition, IFN-exo and miR-106b-3p mimic treatment inhibited the expression of HBV DNA and HBsAg in Huh7- B3 more strongly than in Huh7-C2. Moreover, IFN-exo down-regulated the expression of MMP9 by 36.6% and 77.2%, and the expression of MMP2 by 16.4% and 31.1% in Huh7- C2 and B3, respectively. Based on these findings, the antiviral effect and migration inhibition of IFN-exo were stronger on HBV- B3 than HBV- C2. This was consistent with clinical reports, claiming that C genotype infections were more likely to develop into HCC and respond poorly to IFNs (Wai et al., 2002).

IFN-α is used as an antiviral adjuvant for the curative treatment of HBV-HCC (Ji et al., 2009; Zhang et al., 2014). Studies have shown that exosomes were important in the delivery of antiviral molecules during IFN-α treatment (Wu et al., 2021). Exosomes contain miRNAs derived from parental cells. IFN-α induced LNPC (such as liver-resident macrophages) derived exosomes transfer the antiviral effects to permissive hepatocytes via miRNAs (Li et al., 2013; Yao et al., 2018; Wu et al., 2021). Using microRNA microarray technology, miR-106b-3p was identified as an early IFN-responsive miRNA in chronic hepatitis B patients (Zhang et al., 2012). MiRNAs regulate HBV replication by targeting viral transcripts, epigenetic mechanisms, and transcriptional regulators. In the present study, increased expression of exosome-derived miR-106b-3p was observed via qRT-PCR in the THP-1 supernatant and cells after IFN-α stimulation. After culturing stimulated macrophage-derived exosomes with Huh-HBV-C2 and B3, miR-106b-3p was significantly up-regulated compared to that of the normal culture group,combined with our previous results that there was an increased tendency of miR-106b-3p in IFN treated HepG2-C2 and HepG2-B3 compared to those untreated cells(data not shown), suggesting that miR-106b-3p was an IFN-responsive miRNA in Huh7 cells. However, the expression of miR-106b-3p was not completely consistent across different diseases. For example, the expression of miR-106b in the serum samples of patients with HCC was reportedly elevated in comparison to non-HCC patients (Ali et al., 2019). Additionally, the expression of miR-106b-3p was elevated in colorectal (Mannavola et al., 2020) and esophageal cancers (Zhu et al., 2021). Interestingly, it has also been reported that the expression of miR-106b-3p in the plasma of patients with cirrhosis was significantly decreased, compared to that of patients with HCC (Moshiri et al., 2018). In addition, decrease in the level of miR-106b-3p was observed at the late senescence of an *in vitro* primary human umbilical vein endothelial cells model (Yentrapalli et al., 2015). Based on the conflicting results of the previous studies and the lack of reports on miR-106b-3p expression in HBV-HCC, the present study examined the expression of miR-106b-3p in different cells. The results showed that miR-106b-3p was significantly elevated in Huh7 HCC cells in comparison to normal hepatic LO2 cells. This difference resembled the elevated expression of miR-106b-3p in serum samples of HCC patients, compared to non-HCC patients (Ali et al., 2019). However, the present study showed that miR-106b-3p was significantly lower in HBV (+)-HCC cells (HepG2.2.15 with genotype D, Huh7-C2 and B3-positive cells) than in Huh7 cells. Moreover, it was significantly increased in the Huh7-C2 and B3 cells co-culture with IFN-exo. Thus, in combination with the down-regulation of HBV DNA and HBsAg expression, as well as the inhibition of cell migration and invasion, suggested that miR-106b-3p had different biological effects in HBV(-)-HCC and HBV(+)-HCC. Moreover, miR-106b-3p was carried by IFN-exo to HBV (+)-HCC hepatocytes to exert the antiviral effects.

MiRNAs promote gene silencing and translational repression by inhibiting the translation of target mRNAs, thereby regulating various molecular biological functions. The results of the present study, validated by online website prediction and a dual-luciferase reporter system, showed that PCGF3 was directly targeted by miR-106b-3p. PCGF3 was reportedly involved in the molecular regulation of disease progression. For example, miR-210-3p regulated the low expression of PCGF3 in lung cancer cells, and the down-regulation of PCGF3 promoted lung cancer development and metastasis (Chen et al., 2021). Another study observed a high expression of PCGF3 in NSCLC (Hu et al., 2021). Given the inconsistencies regarding the expression of PCGF3 in cancer and the lack of reports on PCGF3 in HBV-related HCC, the present study analyzed the data of HCC patients in the UALCAN online database. Firstly, the results showed that PCGF3 expression was elevated in HCC patients, and this was associated with a poorer prognosis among HCC patients. Meanwhile, the results of qRT-PCR analysis in the present study showed that the expression of PCGF3 was significantly higher in Huh7 HCC cells and HepG2.2.15 cells than in normal hepatic LO2 cells. Secondly, the expression of PCGF3 was lower in Huh7-C2 and B3-positive cells than in Huh7 cells. Lastly, the expression of PCGF3 in the tumor tissues of patients with HCC, specifically HBV (+)-HCC, was significantly higher than that of paracancerous tissues. These findings suggested an association between PCGF3 and HBV-related HCC malignant disease. Previous studies have shown that chronic HBeAg infection complicated the disease by causing hepatitis, fibrosis progression, cirrhosis, and HCC reactivation (Deng et al., 2020; Koffas et al., 2021). The incidence of HCC was significantly higher in HBeAg-negative patients than in HBeAg-positive patients in the indeterminate phase (Liu et al., 2023). Even after seven years of ETV treatment, HBeAg-negative patients with the HBV genotype C were more likely to progress to HCC than HBeAg-positive patients (Chang et al., 2023), and male HBeAg-negative patients were more likely to progress to HCC (Chang et al., 2023). So, long-term treatment remains necessary for HBeAg-negative chronic hepatitis B patients (Vlachogiannakos and Papatheodoridis, 2014). The results of the present study showed that the expression of PCGF3 was significantly higher in HBeAg-negative tumor tissues compared to HBeAg-positive tissues, and among HBV-related HCC patients, there were more male patients than female patients. However, the relationship between the expression of PCGF3 and the malignant progression of HBV (+)-HCC with different HBeAg statuses needs further investigation.

The abnormal up-regulation of the PI3K/AKT pathway was reportedly associated with numerous tumors (Vasan and Cantley, 2022; Zhang et al., 2023). The activation of the PI3K/AKT signaling pathway in HCC cells significantly up-regulates the expression of MMP2 and MMP9 proteins, thereby promoting HCC cell migration (Tang et al., 2022). The present study also showed that p-PI3K/PI3K and p-AKT/AKT were more significantly elevated in Huh7- and B3 cells than in Huh7 cells. Furthermore, the MMP2/9 protein expression levels were also significantly elevated. The pathway inhibitor treatment significantly down-regulated the expression of p-PI3K/PI3K, p-AKT/AKT, and MMP2/9. Based on this finding, the HBV (+) - PI3K/AKT signaling pathway was activated in Huh7 cells, and inhibition of the PI3K/AKT signaling pathway in HBV-related HCC impeded the migration and invasion of HCC cells. It was reported that PCGF3 expression in lung cancer cell lines positively regulated PI3K/AKT pathway activity (Hu et al., 2021). In breast cancer, miR-106b-3p induces the migration, invasion, and proliferation of breast epithelial cells, and p-AKT activity is enhanced after transfection with miR-106b-3p mimics. This indicates that miR-106b promotes the progression of breast cancer cells by activating the PI3K/AKT pathway (Li et al., 2017). However, the mechanism behind the mediation of the PCGF3 function by miR-106b-3p in HBV-HCC has not been elucidated. In the present study, after the co-culture of IFN-exo or miR-106b-3p-mimic, PI3K/AKT pathway inhibitor and siRNAs-PCGF3 with Huh7-C2 and B3, the expressions of PCGF3, p-PI3K/PI3K and p-AKT/AKT in cells were significantly reduced, compared to those before the treatment. The proliferation, migration, and invasion of the cells were inhibited. Thus, we hypothesized that IFN-exo or miR-106b-3p regulated the malignant biological functions of cancer cells by reducing the PCGF3 expression and inhibiting PI3K/AKT signaling pathway activity in HBV (+)-HCC. These ultimately inhibit the occurrence and development of HBV-HCC.

## Conclusion

In summary, by observing the significant up-regulation of miR-106b-3p after the co-culture of IFN-exo with Huh-HBV-C2 and B3, this study confirmed that miR-106b-3p was an IFN-responsive miRNA. Moreover, IFN inhibited viral replication and viral antigen expression by carrying miR-106b-3p via macrophage-derived exosomes to HBV-positive hepatocytes. The expression of miR-106b-3p was lower in HBV (+) cells than in HBV (-) cells, and higher in Huh7 cells than in normal cells. Therefore, miR-106b-3p might exhibit different biological effects in HBV (-)-HCC and HBV (+)-HCC, which should be considered when miR-106b-3p is used as a target for the prevention and treatment of HCC. The high expression of PCGF3 in HCC cells and HBV-HCC tumor tissues was associated with a poorer prognosis among HCC patients. IFN-induced macrophage-derived exosomes and miR-106b-3p mimic inhibited Huh7- C2/B3 proliferation, migration, and invasion by down-regulating the PCGF3 and the activity of downstream PI3K/AKT signaling pathway. Based on these findings, miR-106b-3p and PCGF3 were important biomarkers in the prevention and treatment of HBV-HCC. However, there were also shortcomings in this experiment. The number of clinical samples collected, specifically the number of patients with HBV (-) HCC and HBsAg (+)/HBeAg (+), was limited. Since the samples did not undergo HBV genotyping in clinical practice, it was not feasible to more precisely correlate the viral genotype with clinical progression. The observation time was insufficient to establish a correlation between the abnormal expression of PCGF3 in the included cases and their prognosis.

## Data Availability

The raw data supporting the conclusions of this article will be made available by the authors, without undue reservation.

## References

[B1] Ali H. E. A. Emam A. A. Zeeneldin A. A. Srour R. Tabashy R. El-Desouky E. D. . (2019). Circulating miR-26a, miR-106b, miR-107 and miR-133b stratify hepatocellular carcinoma patients according to their response to transarterial chemoembolization. Clin. Biochem. 65, 45–52. doi: 10.1016/j.clinbiochem.2019.01.002, PMID: 30653948 PMC6397777

[B2] Almeida M. Pintacuda G. Masui O. Koseki Y. Gdula M. Cerase A. . (2017). PCGF3/5-PRC1 initiates Polycomb recruitment in X chromosome inactivation. Sci. (New York NY). 356, 1081–1084. doi: 10.1126/science.aal2512, PMID: 28596365 PMC6522364

[B3] Araujo N. M. (2015). Hepatitis B virus intergenotypic recombinants worldwide: An overview. Infect. Genet. Evol. 36, 500–510. doi: 10.1016/j.meegid.2015.08.024, PMID: 26299884

[B4] Bello K. E. Mat Jusoh T. N. A. Irekeola A. A. Abu N. Mohd Amin N. A. Z. Mustaffa N. . (2023). A recent prevalence of hepatitis B virus (HBV) genotypes and subtypes in Asia: A systematic review and meta-analysis. Healthc. (Basel Switzerland) 11, 1011. doi: 10.3390/healthcare11071011, PMID: 37046937 PMC10094200

[B5] Chang X. Li Y. Sun C. Li X. Du W. Shang Q. . (2023). High-risk population of progressive hepatic fibrosis in chronic hepatitis B patients on antiviral therapy. J. gastroenterol. 58, 481–493. doi: 10.1007/s00535-023-01970-3, PMID: 36928343

[B6] Chen J. Li L. Yin Q. Shen T. (2023). A review of epidemiology and clinical relevance of Hepatitis B virus genotypes and subgenotypes. Clinics Res. Hepatol. gastroenterol. 47, 102180. doi: 10.1016/j.clinre.2023.102180, PMID: 37479136

[B7] Chen Q. Zhang H. Zhang J. Shen L. Yang J. Wang Y. . (2021). miR-210-3p promotes lung cancer development and progression by modulating USF1 and PCGF3. OncoTargets Ther. 14, 3687–3700. doi: 10.2147/OTT.S288788, PMID: 34140779 PMC8203303

[B8] Deng D. L. Jiang J. N. Su M. H. Wang R. M. Zang W. W. Ling X. Z. . (2020). Liver histological status and clinic outcome in HBeAg-negative chronic hepatitis B with low viral load. Zhonghua gan zang bing za zhi = Zhonghua ganzangbing zazhi = Chin. J. hepatol. 28, 1013–1017. doi: 10.3760/cma.j.cn501113-20201028-00584, PMID: 34865348 PMC12769375

[B9] Elizalde M. M. Tadey L. Mammana L. Quarleri J. F. Campos R. H. Flichman D. M. (2021). Biological characterization of hepatitis B virus genotypes: their role in viral replication and antigen expression. Front. Microbiol. 12, 758613. doi: 10.3389/fmicb.2021.758613, PMID: 34803982 PMC8600256

[B10] Fernandes da Silva C. Keeshan A. Cooper C. (2023). Hepatitis B virus genotypes influence clinical outcomes: A review. Can. liver J. 6, 347–352. doi: 10.3138/canlivj-2023-0003, PMID: 38020195 PMC10652982

[B11] Hadziyannis S. J. (2011). Natural history of chronic hepatitis B in Euro-Mediterranean and African countries. J. hepatol. 55, 183–191. doi: 10.1016/j.jhep.2010.12.030, PMID: 21238520

[B12] Hu Y. Cheng Y. Jiang X. Zhang Y. Wang H. Ren H. . (2021). PCGF3 promotes the proliferation and migration of non-small cell lung cancer cells via the PI3K/AKT signaling pathway. Exp. Cell Res. 400, 112496. doi: 10.1016/j.yexcr.2021.112496, PMID: 33485844

[B13] Ji J. Shi J. Budhu A. Yu Z. Forgues M. Roessler S. . (2009). MicroRNA expression, survival, and response to interferon in liver cancer. New Engl. J. Med. 361, 1437–1447. doi: 10.1056/NEJMoa0901282, PMID: 19812400 PMC2786938

[B14] Kao J. H. (2011). Molecular epidemiology of hepatitis B virus. Korean J. Internal Med. 26, 255–261. doi: 10.3904/kjim.2011.26.3.255, PMID: 22016585 PMC3192197

[B15] Koffas A. Kumar M. Gill U. S. Jindal A. Kennedy P. T. F. Sarin S. K. (2021). Chronic hepatitis B: the demise of the ‘inactive carrier’ phase. Hepatol. Int. 15, 290–300. doi: 10.1007/s12072-021-10137-2, PMID: 33638770

[B16] Komoll R. M. Hu Q. Olarewaju O. von Döhlen L. Yuan Q. Xie Y. . (2021). MicroRNA-342-3p is a potent tumour suppressor in hepatocellular carcinoma. J. hepatol. 74, 122–134. doi: 10.1016/j.jhep.2020.07.039, PMID: 32738449

[B17] Li J. Liu K. Liu Y. Xu Y. Zhang F. Yang H. . (2013). Exosomes mediate the cell-to-cell transmission of IFN-α-induced antiviral activity. Nat. Immunol. 14, 793–803. doi: 10.1038/ni.2647, PMID: 23832071

[B18] Li N. Miao Y. Shan Y. Liu B. Li Y. Zhao L. . (2017). MiR-106b and miR-93 regulate cell progression by suppression of PTEN via PI3K/Akt pathway in breast cancer. Cell Death dis. 8, e2796. doi: 10.1038/cddis.2017.119, PMID: 28518139 PMC5520687

[B19] Liao H. Li X. Liu Y. Xu Z. Huang P. Nian X. . (2017). Intergenotype recombinant analysis of full-length hepatitis B virus genomes from 516 Chinese patients with different illness categories. J. Med. virol. 89, 139–145. doi: 10.1002/jmv.24609, PMID: 27328656

[B20] Lin Q. Zhou C. R. Bai M. J. Zhu D. Chen J. W. Wang H. F. . (2020). Exosome-mediated miRNA delivery promotes liver cancer EMT and metastasis. Am. J. Trans. Res. 12, 1080–1095. PMC7137059 32269736

[B21] Liu B. Yang J. X. Yan L. Zhuang H. Li T. (2018). Novel HBV recombinants between genotypes B and C in 3’-terminal reverse transcriptase (RT) sequences are associated with enhanced viral DNA load, higher RT point mutation rates and place of birth among Chinese patients. Infect. Genet. evol.: J. Mol. Epidemiol. evolution. Genet. Infect. dis. 57, 26–35. doi: 10.1016/j.meegid.2017.10.023, PMID: 29111272

[B22] Liu H. Liu Y. Sun P. Leng K. Xu Y. Mei L. . (2020). Colorectal cancer-derived exosomal miR-106b-3p promotes metastasis by down-regulating DLC-1 expression. Clin. Sci. (London England: 1979). 134, 419–434. doi: 10.1042/CS20191087, PMID: 32065214

[B23] Liu Z. Zhang Y. Xu M. Li X. Zhang Z. (2021). Distribution of hepatitis B virus genotypes and subgenotypes: A meta-analysis. Medicine 100, e27941. doi: 10.1097/MD.0000000000027941, PMID: 34918643 PMC8678021

[B24] Liu M. Zhao T. Zhang Y. Zhang A. M. Geng J. Xia X. (2023). The incidence of hepatocellular carcinoma and clearance of hepatitis B surface for CHB patients in the indeterminate phase: a systematic review and meta-analysis. Front. Cell. infect. Microbiol. 13, 1226755. doi: 10.3389/fcimb.2023.1226755, PMID: 37771696 PMC10523783

[B25] Lu Z. M. Lin Y. F. Jiang L. Chen L. S. Luo X. N. Song X. H. . (2014). Micro-ribonucleic acid expression profiling and bioinformatic target gene analyses in laryngeal carcinoma. OncoTargets Ther. 7, 525–533. doi: 10.2147/OTT, PMID: 24741319 PMC3983076

[B26] Mannavola F. Pezzicoli G. Tucci M. (2020). DLC-1 down-regulation via exosomal miR-106b-3p exchange promotes CRC metastasis by the epithelial-to-mesenchymal transition. Clin. Sci. (London England: 1979). 134, 955–959. doi: 10.1042/CS20200181, PMID: 32313957

[B27] Moshiri F. Salvi A. Gramantieri L. Sangiovanni A. Guerriero P. De Petro G. . (2018). Circulating miR-106b-3p, miR-101-3p and miR-1246 as diagnostic biomarkers of hepatocellular carcinoma. Oncotarget 9, 15350–15364. doi: 10.18632/oncotarget.v9i20, PMID: 29632649 PMC5880609

[B28] Nurainy N. Muljono D. H. Sudoyo H. Marzuki S. (2008). Genetic study of hepatitis B virus in Indonesia reveals a new subgenotype of genotype B in east Nusa Tenggara. Arch. virol. 153, 1057–1065. doi: 10.1007/s00705-008-0092-z, PMID: 18463783

[B29] Qian Z. Jianqiong W. Hongmei L. Rong Z. Li L. Jinping Z. . (2015). Distribution and epidemiologic trends of HBV genotypes and subtypes in 14 countries neighboring China. Hepatitis month. 15, e24422. doi: 10.5812/hepatmon PMC4451280 26045702

[B30] Qiao G. Dai C. He Y. Shi J. Xu C. (2019). Effects of miR−106b−3p on cell proliferation and epithelial−mesenchymal transition, and targeting of ZNRF3 in esophageal squamous cell carcinoma. Int. J. Mol. Med. 43, 1817–1829. doi: 10.3892/ijmm, PMID: 30816445 PMC6414160

[B31] Raab-Traub N. Dittmer D. P. (2017). Viral effects on the content and function of extracellular vesicles. Nat. Rev. Microbiol. 15, 559–572. doi: 10.1038/nrmicro.2017.60, PMID: 28649136 PMC5555775

[B32] Sant’Anna T. B. Araujo N. M. (2023). Hepatitis B virus genotype D: an overview of molecular epidemiology, evolutionary history, and clinical characteristics. Microorganisms 11, 1011. doi: 10.3390/microorganisms11051101, PMID: 37317074 PMC10221421

[B33] Shi Y. H. (2012). Correlation between hepatitis B virus genotypes and clinical outcomes. Japan. J. Infect. dis. 65, 476–482. doi: 10.7883/yoken.65.476, PMID: 23183198

[B34] Tang Y. T. Huang Y. Y. Zheng L. Qin S. H. Xu X. P. An T. X. . (2017). Comparison of isolation methods of exosomes and exosomal RNA from cell culture medium and serum. Int. J. Mol. Med. 40, 834–844. doi: 10.3892/ijmm.2017.3080, PMID: 28737826 PMC5548045

[B35] Tang Z. Zhao P. Zhang W. Zhang Q. Zhao M. Tan H. (2022). SALL4 activates PI3K/AKT signaling pathway through targeting PTEN, thus facilitating migration, invasion and proliferation of hepatocellular carcinoma cells. Aging 14, 10081–10092. doi: 10.18632/aging.v14i24, PMID: 36575044 PMC9831741

[B36] Vasan N. Cantley L. C. (2022). At a crossroads: how to translate the roles of PI3K in oncogenic and metabolic signalling into improvements in cancer therapy. Nat. Rev. Clin. Oncol. 19, 471–485. doi: 10.1038/s41571-022-00633-1, PMID: 35484287 PMC11215755

[B37] Vlachogiannakos J. Papatheodoridis G. V. (2014). HBeAg-negative chronic hepatitis B: why do I treat my patients with pegylated interferon-alfa? Liver Int. 34 Suppl 1, 127–132. doi: 10.1111/liv.12404, PMID: 24373089

[B38] Wai C. T. Chu C. J. Hussain M. Lok A. S. (2002). HBV genotype B is associated with better response to interferon therapy in HBeAg(+) chronic hepatitis than genotype C. Hepatol. (Baltimore Md). 36, 1425–1430. doi: 10.1053/jhep.2002.37139, PMID: 12447868

[B39] Wang W. Shu Y. Bao H. Zhao W. Wang W. Wang Q. . (2019). Genotypes and hot spot mutations of hepatitis B virus in Northwest Chinese population and its correlation with diseases progression. BioMed. Res. Int. 2019, 3890962. doi: 10.1155/2019/3890962, PMID: 31886206 PMC6925797

[B40] Wu W. Wu D. Yan W. Wang Y. You J. Wan X. . (2021). Interferon-induced macrophage-derived exosomes mediate antiviral activity against hepatitis B virus through miR-574-5p. J. Infect. dis. 223, 686–698. doi: 10.1093/infdis/jiaa399, PMID: 32663850

[B41] Yang Y. Liu K. Zhou W. Dai S. (2023). Exosomes from Ub−HBcAg−overexpressing dendritic cells induce T−lymphocyte differentiation and enhance cytotoxic T−lymphocyte activity. Exp. Ther. Med. 25, 167. doi: 10.3892/etm, PMID: 36936705 PMC10015322

[B42] Yao Z. Jia X. Megger D. A. Chen J. Liu Y. Li J. . (2019). Label-free proteomic analysis of exosomes secreted from THP-1-derived macrophages treated with IFN-α Identifies antiviral proteins enriched in exosomes. J. Proteome Res. 18, 855–864. doi: 10.1021/acs.jproteome.8b00514, PMID: 30550287

[B43] Yao Z. Qiao Y. Li X. Chen J. Ding J. Bai L. . (2018). Exosomes exploit the virus entry machinery and pathway to transmit alpha interferon-induced antiviral activity. J. Virol. 92, 1578-18. doi: 10.1128/JVI.01578-18, PMID: 30282711 PMC6258946

[B44] Yentrapalli R. Azimzadeh O. Kraemer A. Malinowsky K. Sarioglu H. Becker K. F. . (2015). Quantitative and integrated proteome and microRNA analysis of endothelial replicative senescence. J. proteom. 126, 12–23. doi: 10.1016/j.jprot.2015.05.023, PMID: 26013412

[B45] Zhang X. Chen C. Wu M. Chen L. Zhang J. Zhang X. . (2012). Plasma microRNA profile as a predictor of early virological response to interferon treatment in chronic hepatitis B patients. Antiviral Ther. 17, 1243–1253. doi: 10.3851/IMP2401, PMID: 22997154

[B46] Zhang W. Song T. Q. Zhang T. Wu Q. Kong D. L. Li Q. . (2014). Adjuvant interferon for early or late recurrence of hepatocellular carcinoma and mortality from hepatocellular carcinoma following curative treatment: A meta-analysis with comparison of different types of hepatitis. Mol. Clin. Oncol. 2, 1125–1134. doi: 10.3892/mco.2014.386, PMID: 25279210 PMC4179807

[B47] Zhang Y. Xiao Y. W. Ma J. X. Wang A. X. (2023). Hydroxysafflor yellow A promotes haCaT cell proliferation and migration by regulating HBEGF/EGFR and PI3K/AKT pathways and circ_0084443. Chin. J. Integr. Med. 30, 213-221. doi: 10.1007/s11655-023-3607-2, PMID: 37688744

[B48] Zhang M. Zhang Z. Imamura M. Osawa M. Teraoka Y. Piotrowski J. . (2021). Infection courses, virological features and IFN-α responses of HBV genotypes in cell culture and animal models. J. hepatol. 75, 1335–1345. doi: 10.1016/j.jhep.2021.07.030, PMID: 34363922 PMC8604785

[B49] Zhu Y. Zhang Y. Li X. Su Y. Wang N. Chen M. . (2021). Downregulation of miR−106b−3p increases sensitivity to cisplatin in esophageal cancer cells by targeting TGM3. Mol. Med. Rep. 23, 471. doi: 10.3892/mmr, PMID: 33899115

