# Correction: IFN-treated macrophage-derived exosomes prevent HBV-HCC migration and invasion via regulating miR-106b-3p/PCGF3/PI3K/AKT signaling axis

**DOI:** 10.3389/fcimb.2026.1793060

**Published:** 2026-03-06

**Authors:** Jing Chen, Qi Yin, Shiheng Xu, Xiaoqing Tan, Yu Liang, Chaohui Chen, Li Li, Tao Zhang, Tao Shen

**Affiliations:** 1 Department of Pulmonary and Critical Care Medicine, Yunnan Provincial Key Laboratory for Clinical Virology, Institute of Basic and Clinical Medicine, The First People’s Hospital of Yunnan Province, Kunming, China; 2 Medical School, Kunming University of Science and Technology, Kunming, China; 3 Faculty of Life Science and Technology, Kunming University of Science and Technology, Kunming, China; 4 Department of Infectious Diseases and Hepatic Disease, Yunnan Province Innovation Team of Intestinal Microecology Related Disease Research and Technological Transformation, The First People’s Hospital of Yunnan Province, Kunming, China

**Keywords:** IFN-α-induced macrophage-derived exosome, miR-106b-3p, PCGF3, HBV-related hepatocellular carcinoma, PI3K/AKT pathway

The title of this article was erroneously given as: “IFN-treated macrophage-derived exosomes prevents HBV-HCC migration and invasion via regulating miR-106b-3p/PCGF3/PI3K/AKT signaling axis”. The correct title of the article is “IFN-treated macrophage-derived exosomes prevent HBV-HCC migration and invasion via regulating miR-106b-3p/PCGF3/PI3K/AKT signaling axis”.

There was a mistake in **Figure 4D** as published. In the published article, the same microscopic field image of the shared Huh7 control well was displayed separately in both the B3 and C2 genotype panels in **Figure 4D**. The corrected **Figure 4D** appears below.

**Figure 4:**
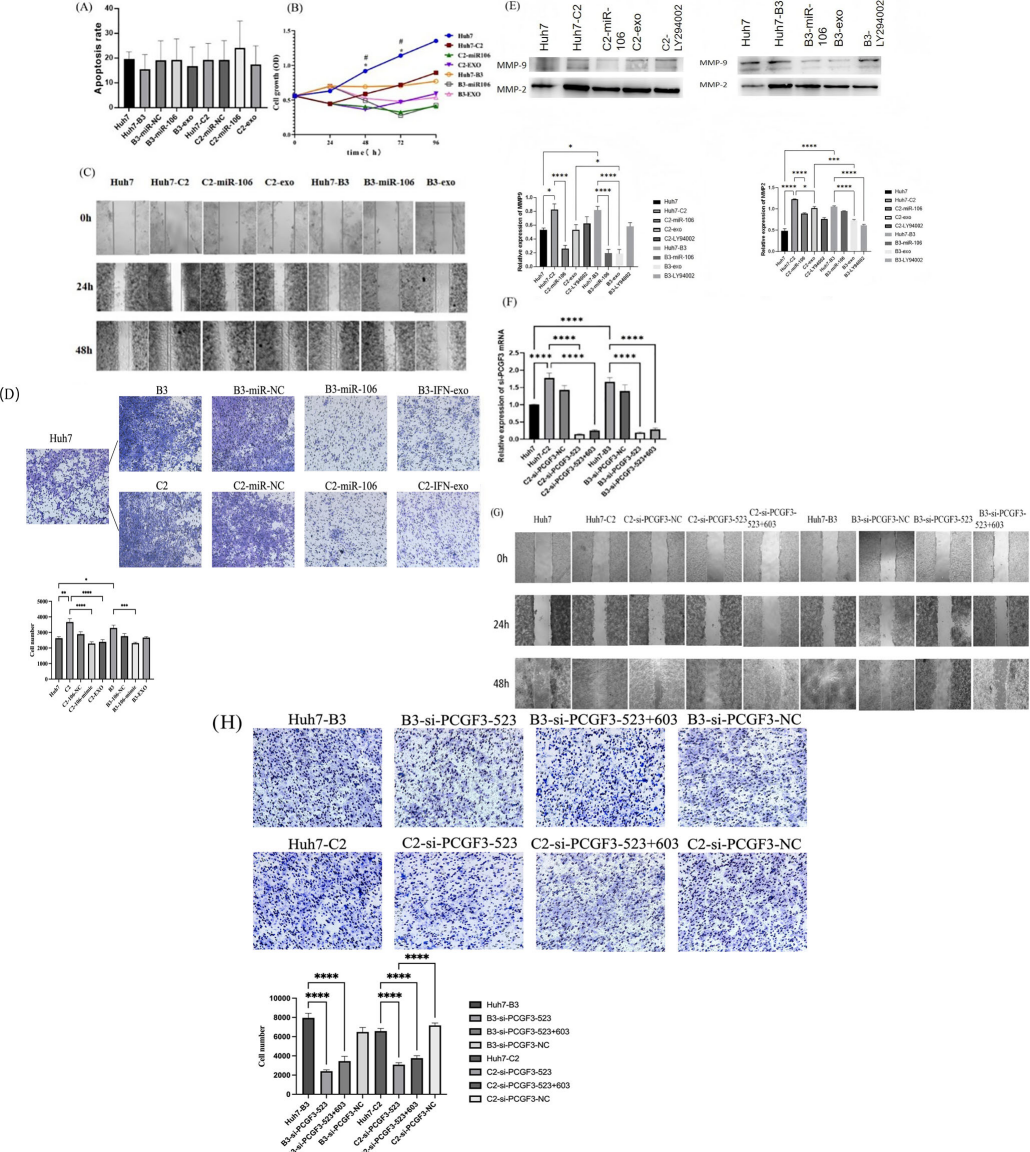
The effect of IFN-α-treated macrophages-derived exosomes (IFN-exo) and miR-106b-3p on PCGF3 expression and Huh7-HBV(+)cells function. **(A)** IFN-exo and miR-106b-3p Annexin V-FITC staining showed no obvious effect on apoptosis of Huh7-C2 and B3 cells untreated or treated with IFN-exo or miR-106b-3p. **(B)** IFN-exo and miR-106b-3p inhibited the proliferation of Huh7-C2 and B3 cells (*represents a statistical difference between Huh7-C2 untreated and treated with miR-106b-3p; # represents a statistical difference between Huh7-B3 untreated and treated with miR-106b-3p). **(C, D)** Scratch wound healing assay and transwell assays revealed that HBV-C2 and B3 positive promoted migration and invasion of Huh7 cells, while IFN-exo and miR-106b-3p abolished the effects of HBV positive on Huh7 cells. **(E)** Western blot analysis indicated that MMP-9 and MMP- 2 were higher expression in HBV-C2 and B3 positive Huh7 cells than Huh7 cells, while IFN-exo, miR-106b-3p and LY294002 reduced the expression of MMP-9 and MMP-2 in HBV-C2 and B3 positive Huh7 cells. (Protein expression was quantified from three independent experiments and normalized to the loading control in each. Loading control bands are not shown.) **(F–H)** siRNAs-PCGF3 knocked-down PCGF3 mRNA expression and inhibited migration and invasion of HBV-C2 and B3 positive Huh7 cells. All data were expressed as mean ± SD. (*, #*P* < 0.05, ** *P* < 0.01, *** *P* < 0.001, ****P < 0.0001).

There was a mistake in **Figure 4E** as published. In the published article, the β-actin loading control image in **Figure 4E** was inappropriately duplicated from **Figure 2D**. The corrected **Figure 4E** appears below.

There was a mistake in **Figure 4H** as published. In the published article, **Figure 4H** inappropriately reused the Huh7 (blank control) image from **Figure 4D**. In addition, the in-figure label for the siRNA was incorrectly given as “siPCGF3-513” instead of “siPCGF3-523”. The corrected **Figure 4H** appears below.

There was a mistake in **Figure 4G** as published. In the published article, the inadvertently presented wound healing assay control images in **Figure 4G** that were duplicated from 4C as pre-established baseline. The corrected **Figure 4G** appears below.

There was a mistake in **Figure 5A** as published. In the published article, the representative AKT band in **Figure 5A** was incorrect. Furthermore, the β-actin loading control image in **Figure 5A** was inappropriately duplicated from **Figure 2D**. The corrected **Figure 5A** appears below.

**Figure 5:**
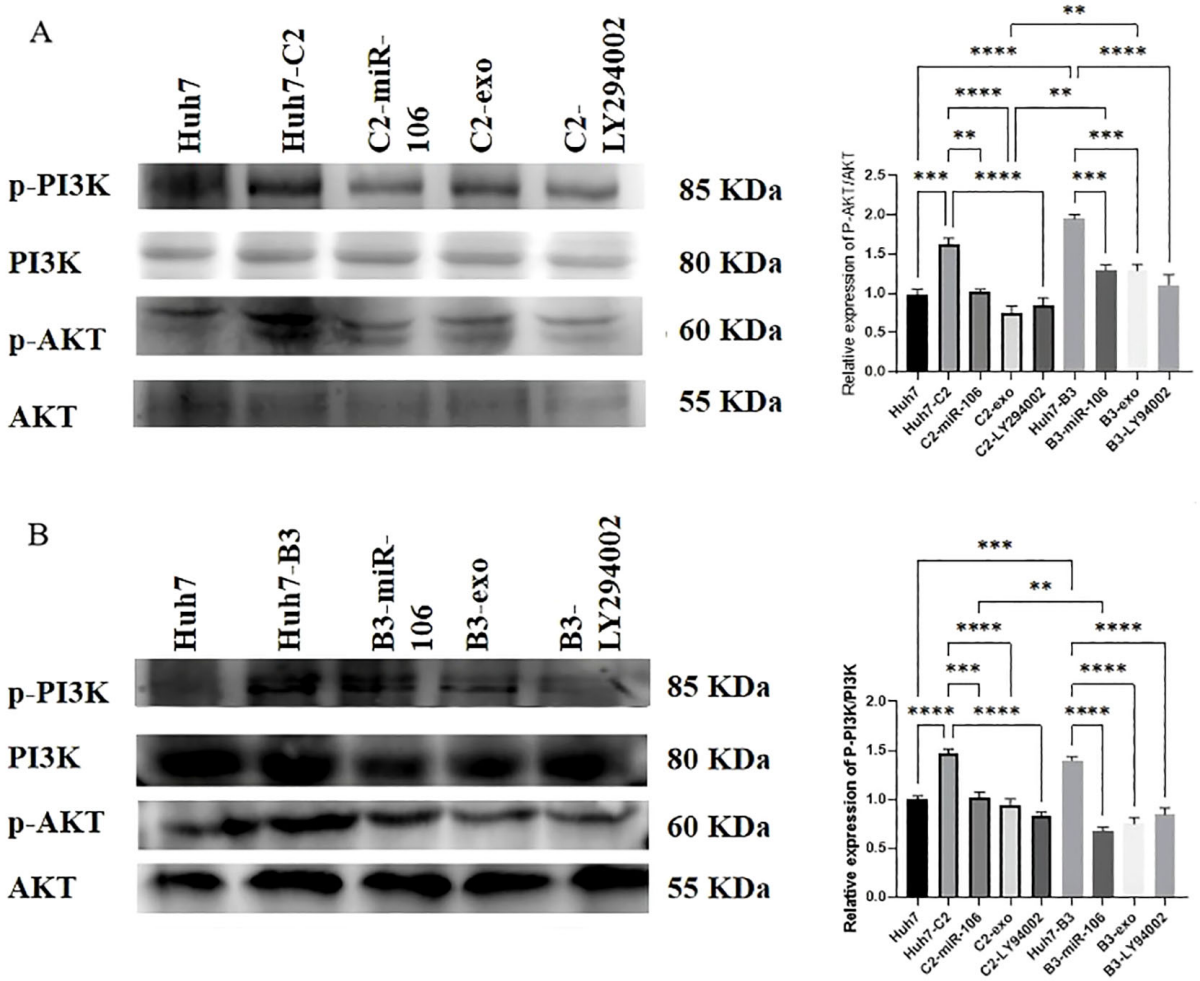
The effect of IFN-a-treated macrophages-derived exosomes (IFN-exo), miR-106b-3p or LY294002 on PI3K-AKT signaling pathway. **(A)** Western blot analysis indicated that the p- AKT/AKT ratio and p-PI3K/PI3K ratio were significantly reduced by treatment with IFN-exo, miR-106b-3p mimic, or LY294002 compared to HBV C2 positive Huh7 cells. **(B)** Western blot analysis indicated that the p-AKT/AKT ratio and p-PI3K/PI3K ratio were significantly reduced by treatment with IFN-exo, miR- 106b-3p mimic, or LY294002 compared to HBV B3 positive Huh7 cells.(** P < 0.01, ***P < 0.001, ****P < 0.0001). (Protein expression was quantified from three independent experiments and normalized to the loading control in each. Loading control bands are not shown).

There was a mistake in **Figure 5B** as published. In the published article, the β-actin loading control image in **Figure 5A** was inappropriately duplicated from **Figure 2D**. The corrected **Figure 5B** appears below.

There was a mistake in the caption of **Figure 2D** as published. In the published article,the legend for **Figure 2D** was incomplete. The corrected caption of **Figure 2** appears below.

“**Figure 2**…//(C, D) miR-106b-3p mimic and IFN-exo can significantly down-regulate the expression of PCGF3 in Huh7 cells with HBV C2 or B3, as determined by qRT-PCR and western blotting All data were expressed as mean ± SD. (*P < 0.05, ** P < 0.01, ***P < 0.001, ****P < 0.0001). (The bar graphs quantifying protein expression (PCGF3 in **Figure 2D**; MMP2/9 in **Figure 4E**; p-PI3K/PI3K, p-AKT/AKT in **Figures 5A, B**) are derived from three independent biological replicates. For each replicate, the band intensity of the target protein was individually normalized to its corresponding loading control signal (β-actin or GAPDH). The normalized values from all three replicates were then pooled and analyzed to generate the final quantitative data presented.)”

There was a mistake in the caption of **Figure 4E** as published. In the original legends for **Figure 4E** was incomplete. The corrected caption of **Figure 4E** appears below.

“**Figure 4**…//(E) Western blot analysis indicated that MMP-9 and MMP- 2 were higher expression in HBV-C2 and B3 positive Huh7 cells than Huh7 cells, while IFN-exo, miR-106b-3p and LY294002 reduced the expression of MMP-9 and MMP-2 in HBV-C2 and B3 positive Huh7 cells. (Protein expression was quantified from three independent experiments and normalized to the loading control in each. Loading control bands are not shown.)//…”

There was a mistake in the caption of **Figure 5** as published. In the original legends for **Figures 5A, B** contained inaccurate descriptions. The corrected caption of **Figure 5** appears below.

“**Figure 5** The effect of IFN-a-treated macrophages-derived exosomes (IFN-exo), miR-106b-3p or LY294002 on PI3K-AKT signaling pathway. (A) Western blot analysis indicated that the p-AKT/AKT ratio and p-PI3K/PI3K ratio were significantly reduced by treatment with IFN-exo, miR-106b-3p mimic, or LY294002 compared to HBV C2 positive Huh7 cells. (B) Western blot analysis indicated that the p-AKT/AKT ratio and p-PI3K/PI3K ratio were significantly reduced by treatment with IFN-exo, miR-106b-3p mimic, or LY294002 compared to HBV B3 positive Huh7 cells.(** P < 0.01, ***P < 0.001, ****P < 0.0001). (Protein expression was quantified from three independent experiments and normalized to the loading control in each. Loading control bands are not shown.)”

A correction has been made to the section [Results, IFN-induced macrophage-derived exosomes or miR-106b-3p inhibits HCC cell growth, migration and invasion via down-regulate PCGF3 expression *in vitro*, line 4 from the bottom in the right column:

“As measured by qRT-PCR and western blot, PCGF3 was effectively down-regulated in Huh7 cells treated with miR-106b-3p mimic or IFN-exo (**Figures 2C, D**)”

A correction has been made to the section Results, IFN-induced macrophage-derived exosomes or miR-106b-3p down-regulates PCGF3 expression and inhibits PI3K/AKT pathway in HCC cell, two consecutive sentences on Page 8, left column (lines 2 and 3 from the bottom:

“The qRT-PCR results showed that after treatment with the pathway inhibitor LY294002, the expression of PCGF3 was significantly reduced in Huh7-B3 cells and 27.4% reduced in Huh7- C2 cells (**Figures 2C**). The western blot results showed that the PCGF3 protein levels, p-AKT/AKT, and p-PI3K were significantly higher in Huh7-C2 and B3 cells than in Huh7 cells (**Figures 2D**, **5A, B**)”

The original version of this article has been updated.

