# Supplementary material for: IFN-treated macrophage-derived exosomes prevents HBV-HCC migration and invasion via regulating miR-106b-3p/PCGF3/PI3K/AKT signaling axis
Source: Front Cell Infect Microbiol. 2024 Oct 28;14:1421195. doi: 10.3389/fcimb.2024.1421195 (PMC11551115; doi:10.3389/fcimb.2024.1421195)
Supplement: Supplementary file 1 [file Table1.docx]

Supplementary table1.Information of antibodies, primers and siRNAs

| Antibodies | Vendor or Source | | Catalog | Source | Working concentration | Molecular weight(kDa) |
| --- | --- | --- | --- | --- | --- | --- |
| PCGF3 | Invitrogen | | AB_10977548 | goat | 1:500 | 28 |
| MMP2 | Cell Signaling Technology | | 40994S | rabbit | 1:1000 | 64,72 |
| MMP9 | Cell Signaling Technology | | 13667S | rabbit | 1:1000 | 84, 92 |
| AKT | Cell Signaling Technology | | 4691 | rabbit | 1:2000 | 55 |
| p-AKT | Cell Signaling Technology | | 4060 | rabbit | 1:1000 | 60 |
| PI3K | Affinity Biosciences | | AF6241 | rabbit | 1:1000 | 80 |
| p-PI3K | Affinity Biosciences | | AF3241 | rabbit | 1:1000 | 85 |
| GAPDH | Cell Signaling Technology | | 97166 | rabbit | 1:1000 | 37 |
| β-actin | Affinity Biosciences | | T0022 | rabbit | 1:1000 | 43 |
| protein marker I | Servicebio | | G2086 |  |  |  |
| HRP-conjugated anti-mouse IgG | Solarbio | | SPA131 | goat | 1:1000-1:10000 |  |
| HRP-conjugated anti-rabit IgG | Solarbio | | SA134 | goat | 1:1000-1:10000 |  |
| Genes | | Forward (5’-3’) | | Reverse(5’-3’) | | |
| PCGF3 | | ccgggagacatcaagggggagac | | tgcggtggtagtcgttgtcctcct | | |
| GAPDH | | gcaccgtcaaggctgagaac | | tggtgaagacgccagtgga | | |
| hsa-miR-106b-3p | | cgccgcactgtgggtact-3' | |  | | |
| U6 | | tggaacgcttcacgaatttgcg-3' | | ggaacgatacagagaagattagc | | |
| Si-PCGF3-NC | | UUCUCCGAACGUGUCACGUTT | | ACGUGACACGUUCGGAGAATT | | |
| Si-PCGF3(987) | | GUCACUAGGUGGAGAUUCATT | | UGAAUCUCCACCUAGUGACTT | | |
| Si-PCGF3(916) | | CCUUUAACGAGCUGGACAUTT | | AUGUCCAGCUCGUUAAAGCTT | | |
| Si-PCGF3(603) | | CAGAGGGAGUUCUAUCACATT | | UGUGAUAGAACUCCCUCUGTT | | |
| Si-PCGF3(523) | | GUACAUCGGUCAUGACAGATT | | UCUGUCAUGACCGAUGUACTT | | |
